# Elevated Chemerin Levels in Pakistani Men: An Interrelation with Metabolic Syndrome Phenotypes

**DOI:** 10.1371/journal.pone.0057113

**Published:** 2013-02-28

**Authors:** Syeda Sadia Fatima, Kiymet Bozaoglu, Rehana Rehman, Faiza Alam, Abdul Shakoor Memon

**Affiliations:** 1 Department of Biological and Biomedical Sciences, Aga Khan University, Karachi, Pakistan; 2 Genomics and Systems Biology, Baker IDI Heart and Diabetes Institute, Melbourne, Victoria, Australia; 3 Department of Physiology, Bahria University Medical and Dental College, Karachi, Pakistan; 4 Department of Physiology, Basic Medical Sciences Institute, Jinnah Postgraduate Medical Centre, Karachi, Pakistan; INRA, France

## Abstract

Chemerin is a novel protein linked to adipocyte differentiation and the development of metabolic imbalances. We sought to examine the relationship of chemerin with metabolic syndrome disturbances including body fat percentage, serum lipid, glucose, insulin levels and body fat percentage in lean and obese volunteers. A cross-sectional study of 90 randomly selected healthy males from Pakistan were divided into three groups as per Body Mass Index (BMI) criteria for South Asian Population. Anthropometric measurements were taken for BMI, waist circumference, hip circumference and body fat percentage, while serum analyses were performed for fasting blood glucose, fasting insulin, fasting lipid profile and serum chemerin. Associations between serum chemerin levels and body fat and other metabolic syndrome parameters were performed using ANOVA and multiple regression analyses. Data was presented as Mean±SD. In all statistical analyses p-values <0.05 were considered significant. Circulating chemerin levels were significantly higher in obese subjects with BMI greater than 25 kg/m^2^ compared with those with a BMI below 25 kg/m^2^ (P = 0.001). Serum chemerin levels were found to be independently and significantly associated with serum levels of cholesterol (P = 0.0160; r = 0.255), fasting glucose (P = 0.002; r = 0.323), HOMA-IR (P = 0.004; r = 0.300) and hip circumference (P = 0.021; r = 0.246). This demonstrates that chemerin levels are associated with obesity and dyslipidemia and may play a role in the development of insulin resistance. This data suggests that chemerin may serve as an independent marker in diagnosing these conditions even before they become clinically symptomatic.

## Introduction

Over the past few years, obesity has become a global epidemic and has emerged as a major health problem associated with an increased risk for cardiovascular disease, diabetes, dyslipidemia and an increased mortality rate [1]–[3]. The popular argument is that the epidemic is due to the changes in the societies’ so called ‘modernization’ leading to over nutrition and a sedentary lifestyle [4].

According to the Asia Pacific Cohort Studies Collaboration [5], the prevalence of obesity ranges from 0.6–4.0% in Asian Indians, where genes, age and sex have been considered as non-modifiable risk factors. A survey of Pakistan data [6] clearly demonstrated that 1% of the Pakistani population was reported to be obese and 5% overweight in people aged 15–24 years of age. Similarly Troiano *et al.* [7] and Jafer *et al*. [8] revealed that according to the Asian specific body mass index (BMI) cutoff value of 23 kg/m^2^, 1 in 4 people in Pakistan over the age of 15 years are overweight or obese.

The key element of obesity is white adipose tissue, which acts as a multifunctional endocrine tissue, regulating adipocyte biology and systemic processes like food intake, nutrient metabolism, insulin sensitivity bone growth, inflammation and reproduction [9], leading to an increased incidence of cardiovascular diseases and Type 2 diabetes [10], [11]. Obesity and physical inactivity are two primary risk factors for the development of hypertension, insulin resistance and dyslipidemia. Serum biomarkers have emerged as important tools for prediction, diagnosis, and risk stratification for patients with obesity related co morbidities [12], [13].

Chemerin, also known as Retinoic Acid Receptor Responder Protein 2 (RARRES 2) is among the newly discovered adipokines. Human pro-chemerin (the inactive form) is synthesized as 163 amino acids with a 20 amino acid hydrophobic signal peptide and is activated through the cleavage of the C terminus by inflammatory and coagulation serine proteases [14]–[16]. The secreted, mature form of chemerin contains 146 amino acids, and has a molecular weight of 16 kDa [16]. Due to its role in adipocyte differentiation and glucose uptake, chemerin is classified as an adipokine [17]–[20]. Chemerin was originally reported to be present in circulation in plasma and serum respectively at 3 and 4.4 nM concentrations in humans and 0.5 and 0. 6 nM concentration in mice [21], [22].

Chemerin has been recently described to be secreted from mature adipocytes and the circulating levels of chemerin in human plasma increased with obesity, which may suggest that chemerin expression may reflect the state of differentiation of adipocytes, adipocyte cell size or total body fat mass [17], [23]. A critical function of chemerin is to regulate adipogenesis and metabolic homeostasis in adipocytes and it may play an important role in macrophage infiltration into adipose tissue [18].

Taken together chemerin is considered to be a marker of adiposity and may have a key role in the development of health consequences in obesity. We therefore hypothesized that circulating chemerin levels have an association with the degree of corpulence. Consequently, this study aimed to examine the relationship between chemerin and body fat percentage, serum lipid, glucose, and insulin levels, in lean and obese healthy volunteers.

## Materials and Methods

We recruited 90 healthy male subjects, between ages 15–65 years, who visited the free body fat screening scheduled at the Department of Physiology, Basic Medical Sciences Institute, Jinnah Post Graduate Medical Centre, Karachi, Pakistan.

### Ethics Statement

The research protocol was approved by the board of advanced studies and research, University of Karachi (BASR No/9088/BMSI) and the Basic Medical Sciences Institute research ethics committee (14/2/11/SSF/BMSI). All clinical investigation was conducted according to the principles expressed in the Declaration of Helsinki. All the participants were volunteers who were explained about the minimal risk research procedure and were asked to complete a verbal and written informed consent.

All subjects included in the study had no previous history or evidence of cardiovascular disease, diabetes, moderate to severe hypertension (resting blood pressure (BP)>170/100 mmHg), dyslipidemia, body weight fluctuation of 5 kg in the recent 6 months, acute infectious disease or chronic inflammatory disease, endocrine disease, cancer or medication usage that could affect cardio metabolic function. Smokers, alcoholics and females were excluded from our study.

The WHO Stepwise Approach to Surveillance (STEPS) [24] protocol was used to measure the waist and hip circumference as previously described. Waist to hip ratio was calculated by dividing the waist circumference by hip circumference. BMI was calculated by dividing weight by height squared (kg/m^2^) [25].

Blood samples were collected in the morning after an overnight fast. Fasting glucose by GOD-PAP (Glucose Oxidase-Phenol-Aminophenazone) method (Merck, France), total cholesterol by Enzymatic Endpoint Method, triglyceride by GPO (Glycerol-3-Phosphate Oxidase-Phenol Aminophenazone) method and HDL-cholesterol levels by CHOD-PAP (Cholesterol Oxidase-Phenol Aminophenazone) method (Randox Laboraties, UK), were measured using a Spectrophotometer(model AE-350, Erma Inc., Tokyo, Japan). LDL-cholesterol levels were calculated using the Friedewald equation [26].

Fasting insulin was measured using an ELISA kit (DIA source Immuno Assay S.A., Belgium). Insulin resistance was calculated using the homeostasis model assessment of insulin resistance (HOMA-IR) index [fasting insulin (units per milliliter) x fasting glucose milligram/deciliter)/405] [27]. The body fat percentage was measured using Diagnostic Scale BG55 (Beurer Germany) through bioelectrical impedance matching/analysis. Serum chemerin levels were measured with an enzyme immunoassay kit (Creative Diagnostics, USA), with a sensitivity of less than 3 pg/ml, using ELISA plate reader equalizer ER 2005, (Eqiupar, Italy).

A descriptive statistical analysis of continuous variables was performed using SPSS (version 11; SPSS Inc., Chicago, IL, USA). Data on continuous variables i.e. biophysical (age, height, weight, BMI, waist circumference, hip circumference, waist hip ratio, body fat, blood pressure etc.) and biochemical (Serum cholesterol, triglycerides, HDL, LDL, fasting blood glucose, fasting insulin and serum chemerin etc.) parameters were calculated as mean ± standard deviation (SD). Statistical comparisons were computed using a student t-test and a one-way analysis of variance (ANOVA) for continuous/quantitative variables, after adjusting for age, and BMI. Pearson’s or Spearman’s coefficient of correlation (r) were used to determine the correlation between serum chemerin levels and lipid profile, fasting blood glucose, insulin and body fat parameters wherever applicable. In all statistical analysis performed p-values <0.05 were considered significant.

## Results

**Figure 1:**
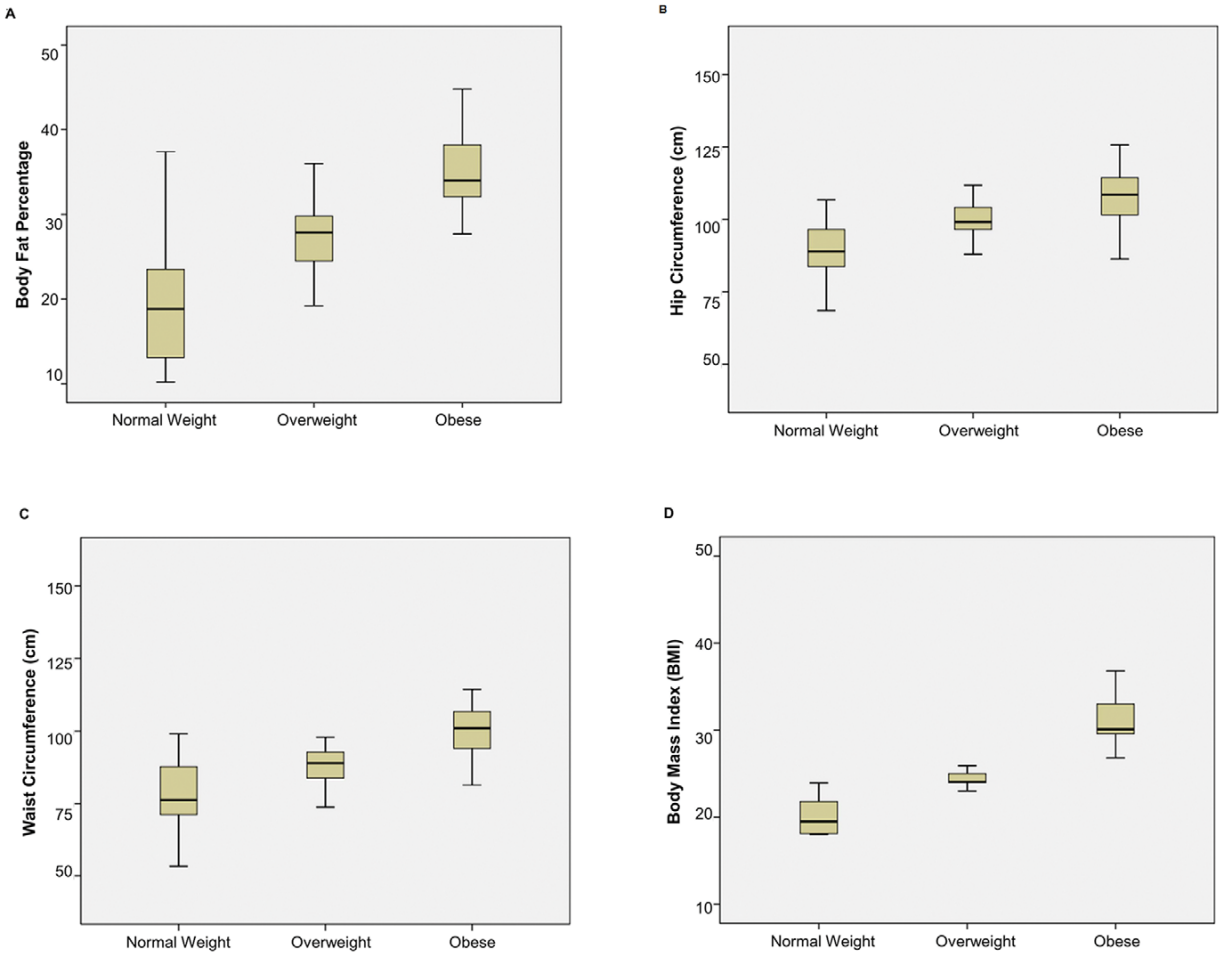
Box-Plot of Anthropometric Parameters of Study Groups. (A) Body fat percentage (B) Hip circumference (C) Waist circumference (D) Body mass index. Significant differences were observed in both overweight (Group B; p<0.05) and obese groups (Group C; p<0.01) compared with normal weight group (Group A; p<0.05). There were 30 subjects per group.

**Figure 2:**
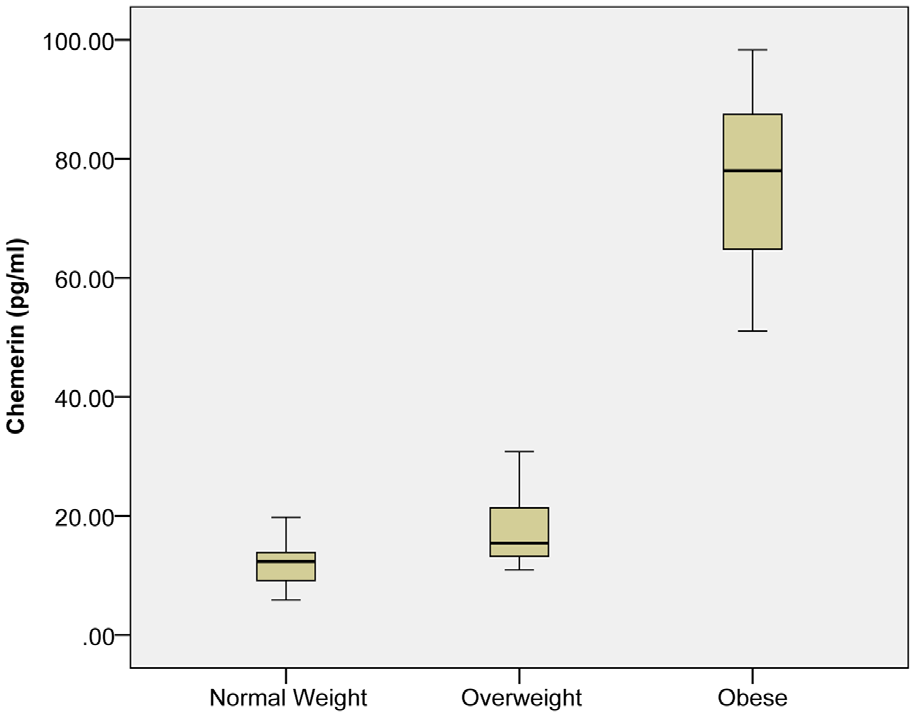
Box-Plot of Circulating Chemerin Levels. Elevated levels of chemerin were observed in obese males compared to normal weight and overweight subjects.

A total of 90 male subjects participated in this study and were divided into three groups according to BMI criteria for South Asian population [28]. Group A were subjects with normal weight (BMI = 18–22.9 kg/m^2^), Group B were overweight (BMI = 23–25 kg/m^2^), Group C were obese (BMI>25 kg/m^2^) subjects. All subjects were age matched and therefore no significant differences were observed between the groups. The biochemical and biophysical parameters of the subjects are outlined in Table 1 and Figure 1. Briefly, no significant changes were observed with height among all groups, but weight portrayed a strong positive correlation with serum chemerin concentrations (P<0.01; r = 0.705). Serum chemerin concentration was significantly increased in the obese group (Group C) (76.4±13.4) as compared to the normal weight group (Group A) (12.0±3.3) and overweight group (Group B) (17.2±6.1) (P = 0.001; (Figure 2). This suggests that the degree of adiposity determines the adipokine level, which in turn may be responsible for metabolic disturbances found in obesity.

**Table 1:** Biophysical and Biochemical Characteristics of the study subjects.

Phenotypes	Normal Weight	Overweight	Obese	P value
	Mean ± SDn = 30	Mean ± SDn = 30	Mean ± SDn = 30	
**Age (Year)**	31.9±15.0	33.3±9.4	38.6±10.4	0.092
95% CI	26.88,38.06	29.79,36.88	34.75, 42.51	–
**Weight (Kg)**	57.9±7.10	71.2±7.11*	88.4±16.2* ^,^ **	0.001
95% CI	55.28,60.60	68.46,73.76	82.35, 94.47	–
**BMI**	20.0±1.92	24.4±0.7*	31.9±4.6* ^,^ **	0.001
95% CI	19.32,20.76	24.10, 24.65	30.23, 33.65	–
**Waist Circumference (cm)**	77.4±11.9	88.2±7.6*	100.4±12.9* ^,^ **	0.001
95% CI	73.63,81.86	85.41, 91.11	95.59,105.22	–
**Hip Circumference (cm)**	88.0±10.5	100.8±6.9*	109.3±11.5* ^,^ **	0.001
95% CI	84.69,91.94	98.19,103.3	104.9,113.5	–
**WHR**	0.88±0.11	0.88±0.07	0.91±0.07	0.247
95% CI	0.83, 0.92	0.85, 0.91	0.88, 0.94	–
**Body Fat (%)**	19.2±7.22	27.3±4.0*	34.9±4.5* ^,^ **	0.001
95% CI	16.89,21.89	25.75, 28.82	33.22, 36.56	–
**Cholesterol (mg/dl)**	144.4±30.4	150.9±39.0	209.1±55.5* ^,^ **	0.001
95% CI	132.69,155.9	135.00,164.19	188.3,229.7	–
**Triglycerides (mg/dl)**	128.1±46.7	142.0±68.6	167.0±56.0* ^,^ **	0.039
95% CI	110.6,145.57	116.26,167.53	146.12,187.9	–
**HDL-C(mg/dl)**	39.5±9.7	37.57±8.6	36.4±6.4	0.353
95% CI	35.32, 42.60	34.51, 40.95	34.00,38.79	–
**LDL-C (mg/dl)**	74.1±30.0	81.6±40.7	134.8±59.0* ^,^ **	0.001
95% CI	63.23, 85.61	66.32, 96.79	112.75,156.8	–
**Fasting glucose (mg/dl)**	83.6±24.11	84.0±18.5	126.9±40.4* ^,^ **	0.001
95% CI	73.38,91.39	77.13, 90.99	111.83,142.02	–
**Fasting Insulin (U/ml)**	19.1±8.4	23.0±10.0	35.4±14.2* ^,^ **	0.001
95% CI	15.95, 22.21	19.24, 26.73	30.14, 40.74	–
**HOMA-IR**	4.1±3.2	4.9±2.8	11.0±6.0* ^,^ **	0.001
95% CI	2.85, 5.25	3.85, 5.99	8.80, 13.26	–
**QIUCKI**	0.31±0.02	0.31±0.03	0.28±0.02* ^,^ **	0.001
95% CI	0.30, 0.32	0.29, 0.31	0.26, 0.28	–
**Chemerin (pg/ml)**	12.0±3.3	17.2±6.1	76.4±13.4* ^,^ **	0.001
95% CI	10.69,13.14	14.93,19.54	56.49,96.39	–

WHR (waist hip ratio), QUICKI (Quantitative check index for insulin sensitivity), HOMA-IR (Homeostatic model of insulin resistance), BMI (Body mass index).

* Statistically significant as compared to control subjects, where p<0.05.

** Statistically significant as compared to overweight subjects, where p<0.01.

Significant changes were observed in the diastolic blood pressure in group B and C as compared to control A (P = 0.001), and a significant positive correlation (P<0.01; r = 0.302) was observed with serum chemerin concentration. Both BMI (P = 0.001; r = 0.769) and total fat percentage (P = 0.001; r = 0.550) were significantly associated with circulating chemerin concentrations. Systolic blood pressure, pulse, temperature and respiratory rate were non-significant in all groups.

Multiple regression analyses were used to assess the associations between chemerin levels and biochemical parameters (Table 2). Briefly, waist circumference (P = 0.001; r = 0.567) and hip circumference (P = 0.001; r = 0.470) showed a positive correlation with serum chemerin concentration, in group B and C as compared to group A. Fasting plasma glucose levels (P = 0.001; r = 0.531), serum insulin level (P = 0.001; r = 0.360), HOMA-IR (P = 0.001; r = 0.524), showed positive correlations, while QUICKI (P = 0.001; r = −0.4), depicted negative correlations with circulating chemerin concentration. Serum cholesterol (P = 0.001; r = 0.575), serum triglyceride levels (P = 0.039; r = 0.230) and LDL-C (P = 0.001; r = 0.487) were significantly higher in group C subjects as compared to group B and A (P = 0.001). While serum levels of HDL-C showed no significance between any of the groups (P = 0.353; r = −0.146), after adjustment for age and BMI, cholesterol (P = 0.0160; r = 0.255), fasting glucose (P = 0.002; r = 0.323), HOMA-IR (P = 0.004; r = 0.300), and hip circumference (P = 0.021; r = 0.246) were found to be independently associated with chemerin levels.

**Table 2:** Correlation of serum chemerin levels with study parameters.

Phenotype	Unadjusted(r)	P value	Adjusted for Age and BMI(r)	P value
**Age (year)**	0.123	0.250	–	–
**BMI**	0.769**	<0.001	–	–
**Waist Circumference (cm)**	0.567**	<0.001	0.022	0.841
**Hip Circumference (cm)**	0.470**	<0.001	0.246*	0.021
**WHR**	0.257*	0.014	0.187*	0.031
**Body Fat (%)**	0.550**	<0.001	0.288*	0.015
**Cholesterol (mg/dl)**	0.575**	<0.001	0.255*	0.016
**Triglycerides (mg/dl)**	0.230*	0.029	0.136	0.207
**HDL-C (mg/dl)**	−0.146	0.169	−0.057	0.596
**LDL-C (mg/dl)**	0.487**	<0.001	0.188	0.080
**Fasting glucose (mg/dl)**	0.531**	<0.001	0.323**	0.002
**Fasting Insulin (U/ml)**	0.360**	<0.001	0.083	0.440
**HOMA-IR**	0.524**	<0.001	0.300**	0.004
**QIUCKI**	−0.440**	<0.001	−0.172	0.110
**Diastolic Blood Pressure (mmHg)**	0.302**	0.004	−0.131	0.223
**Systolic Blood Pressure (mmHg)**	0.128	0.228	−0.022	0.841

WHR (waist hip ratio), QUICKI (Quantitative check index for insulin sensitivity), HOMA-IR (Homeostatic model of insulin resistance), BMI (Body mass index), r = correlate on coefficient.

* Significant correlation whereby p<0.05.

** Significant correlation whereby p<0.01.

## Discussion

Adipose tissue not only acts as an energy reservoir but also acts like a remote endocrine organ and has an important role in regulating energy homeostasis and metabolism by communicating with liver, skeletal muscle, and the brain [29], [30]. Recently, the new adipokine chemerin has been characterized to be associated with increased white cell mass, and obesity induced inflammation in adipose tissue [31], [32]. Conde and his group [33] have identified a novel role of chemerin stating that chemerin along with other adipokines such as leptin, adiponectin, lipocalin and serum amyloid A3 are also expressed in non-adipose tissues especially the chondrocytes. This is plausible that these adipokines may then regulate chondrocyte biology and bone development, apart from their role in metabolic diseases. This may be reflected as altered adipokine secretion which in turn promotes chronic inflammatory state in rheumatic disease, including cartilage, synovium, bone and various immune cells [34]. Chemerin, is a 16 kDa protein secreted in an inactive form as prochemerin and is activated through the cleavage of the C-terminus by inflammatory and coagulation serine proteases [14]. In mice, chemerin mRNA is highly expressed in white adipose tissue, liver and lung, and chemerin knockdown impairs differentiation of adipocytes, reduces the expression of GLUT4 while increasing expression of IL-6 and insulin receptor and reduces lipolysis [17], [18]. Furthermore, in humans, plasma chemerin levels were significantly associated with obesity parameters in several different populations [17], [22], [35]–[37]. Collectively, this suggests that chemerin may play a role in the metabolic function of mature adipocytes.

Our results identified a significant increase in serum chemerin levels in obese subjects, related to the amount of total percentage of body fat. Chemerin prominently revealed a strong positive correlation with increasing weight, BMI, waist and hip circumference and body fat percentage, in a healthy population. This data is consistent with previously published reports whereby gene expressions as well as protein levels of chemerin and chemerin’s receptor, CMKLR1 were significantly increased in mice fed a high fat diet [17], [37]. A marked increase in chemerin concentration was also observed in obese individuals, linking with subcutaneous and omental adipose tissue, BMI, WHR, and skinfold thickness [38]–[40]. These studies are in agreement with the findings of our study.

We also established that the fasting glucose and insulin levels were raised in obese subjects despite having no complaints or symptoms of diabetes, these levels along with HOMA-IR showed a strong positive correlation with chemerin, while QUICKI had a negative correlation. Previous studies have shown significant positive correlations between chemerin levels and fasting blood glucose, fasting insulin, HOMA-IR, HbA1c, independent of age and BMI were observed in subjects with type II diabetes compared to normal glycemic subjects [39], [41], [42]. These studies are consistent with our data, however it is important to note that our data only incorporated and reported findings in healthy subjects whereas other studies performed these associations in diabetic humans and mice. Taken together, this may deduce a role for chemerin as a screening tool in individuals who have higher visceral fat accumulation contrasting to being in the range of normal weight category, which is seen commonly in Asian population. This is important, since BMI has some limitations as a measure of adiposity across populations [43].

Fasting glucose levels and HOMA-IR remained independently associated with chemerin levels even after adjustment for age and BMI. Thus we can confidently conclude that chemerin levels may affect glucose homeostasis and therefore may lead to the development of insulin resistance [17]. These elevated serum chemerin levels observed in human and mice suggest that chemerin might also influence the dysregulation of glucose metabolism that often occurs with obesity, via the induction of insulin resistance especially in skeletal muscle. Recent studies demonstrated that chemerin induces insulin resistance in skeletal muscle by inhibiting insulin-stimulated Akt1 phosphorylation and activating the 5 AMP-activated protein kinase (AMPK) [42], glycogen synthase kinase 3 phosphorylation, and glucose uptake [44].This decreased uptake could either be a result of down regulation of glucose transporters or competitive inhibition of chemerin to these receptors. Further studies are required to evaluate these mechanisms.

Here, we demonstrated that in a healthy group of subjects, serum cholesterol, triglyceride and LDL-C were significantly higher in obese versus lean and overweight subjects, and that a strong positive correlation of cholesterol, triglycerides and LDL-C exists with chemerin, while serum levels of HDL-C showed non-significant negative correlation with chemerin concentration. These findings are comparable to various studies [38], [45], [46] with a slight variation in our results being reported in healthy subjects as opposed to diabetic subjects. Positive correlations between triglycerides, HDL-C and systemic chemerin was also identified in normal glycemic patients by Bozaoglu *et al.* [35] but no correlations were observed by Becker *et al.* [42], Weigert *et al*. [47] & Landgraf *et al.* [48]. These associations were lost after adjusting data for age and BMI, but cholesterol levels showed a positive correlation with chemerin even after that. These observations suggest that chemerin could also be a regulator of fat metabolism.

A strong positive correlation with diastolic blood pressure was observed in this study. Comparable results were observed suggesting that serum chemerin levels were significantly associated with systolic hypertension even after adjusting for anthropometric variables [17], [41], [48], [49] however no correlation of blood pressure with chemerin was identified by and Hah *et al*. [46]. We clearly demonstrated that serum chemerin levels are associated with parameters of metabolic syndrome including hypertension regardless of presence of other metabolic syndrome components, signifying that chemerin may also be a distinctive regulator of blood pressure because of its significant correlation with diastolic pressure. Moreover, it has been shown that chemerin is structurally similar to Bradykinin which is also involved in blood pressure regulation [50].This effect of chemerin on blood pressure may also relate to its high expression by kidneys, which is the primary regulator of blood pressure [22], [42].

Studies have reported that chemerin demonstrated no gender dimorphism in a cohort of type II diabetic patients [47] in 55 healthy volunteers [22] & in non-obese chronic hepatitis patients [51]. In contrast Tan *et al*. [38] and Bozaoglu *et al*. [17], [23] found that chemerin levels increased with age and that there appears to be no diurnal variation in chemerin levels. However we found no significant association between chemerin levels and age, similar to a study in a group of pregnant females [52]. While Landgraf *et al*. [48] reported no correlation between sex and chemerin levels but a negative relation with age and circulating levels especially in boys. These differences may be due to the variations in culture and environmental settings.

Chemerin may provide an exciting connection between obesity, inflammation and obesity related pathophysiological changes in humans. Even though a relationship of elevated serum levels in obesity and metabolic syndrome has been suggested, further studies are required that will provide valuable insight to verifying the function of chemerin and determine whether excess chemerin increases adiposity and deranges metabolic function or whether elevated chemerin levels are a consequence or compensatory response during and following the development of obesity and its co morbidities which may lead to the potential therapeutic role of chemerin to these prevalent disorders.

### Conclusion

Obesity and physical inactivity are two primary risk factors for the development of hypertension, insulin resistance and dyslipidemia. Significant associations between chemerin levels and various phenotypes of the metabolic syndrome were observed in our population. These findings suggest that chemerin may provide an interesting screening or diagnostic tool for obesity and its complications in humans and may represent a widespread phenotypic relationship that is not population-specific. Whether chemerin is a cause or consequence of these disorders remains to be elucidated and requires further investigation.
